# Comparative development of human filariae *Loa loa, Onchocerca volvulus* and *Mansonella perstans* in immunocompromised mouse strains

**DOI:** 10.3389/fitd.2024.1293632

**Published:** 2024-04-15

**Authors:** Valerine C. Chunda, Fanny Fri Fombad, Chi Anizette Kien, Rene Ebai, Frederick Esofi, Anna Ning Ntuh, Emmanuel Ouam, Narcisse Victor Tchamatchoua Gandjui, Relindis Ekanya, Franck Nietcho, Lucy Cho Nchang, Chefor Magha, Abdel Jelil Njouendou, Peter Enyong, Achim Hoerauf, Samuel Wanji, Manuel Ritter

**Affiliations:** 1 Parasite and Vector Research Unit (PAVRU), Department of Microbiology and Parasitology, University of Buea, Buea, Cameroon; 2 Research Foundation for Tropical Diseases and the Environment (REFOTDE), Buea, Cameroon; 3 Institute of Medical Microbiology, Immunology and Parasitology (IMMIP), University Hospital Bonn (UKB), Bonn, Germany; 4 German Centre for Infection Research (DZIF), Partner Site Bonn-Cologne, Bonn, Germany; 5 German-West African Centre for Global Health and Pandemic Prevention (G-WAC), Partner Site Bonn, Bonn, Germany

**Keywords:** *Mansonella perstans*, *Loa loa*, *Onchocerca volvulus*, murine models of human filariasis, immunocompromised mice

## Abstract

**Introduction:**

Mouse models of human filarial infections are not only urgently needed to investigate the biology of the nematodes and their modulation of the host’s immunity, but will also provide a platform to screen and test novel anti-filarial drugs. Recently, murine *Loa loa* infection models have been stablished using immunocompromised mouse strains, whereas murine *Mansonella perstans* infections have not been implemented until now.

**Methods:**

Therefore, we aim to establish experimental *M. perstans* infections using the immunocompromised mouse strains RAG2IL-2Rγ^−/−^ (lack B, T and natural killer cells), IL-4Rα/IL-5^−/−^ (impaired IL-4/5 signalling and eosinophil activation) and NOD.Cg-Prkdc^scid^Il2rg^tm1Wj^ l/SzJ (NOD scid gamma, NSG) BALB/c mice (lack mature lymphocytes) through subcutaneous (s.c.) or intraperitoneal (i.p.) inoculation of infective stage 3 larvae (L3) isolated from engorged vectors.

**Results:**

In total, 145 immunocompromised mice have been inoculated with 3,250 *M. perstans*, 3,337 *O. volvulus*, and 2,720 *Loa loa* L3 to comparatively analyse which immunocompromised mouse strain is susceptible to human filarial infections. Whereas, no *M. perstans* and *O. volvulus* L3 could be recovered upon 2-63 days post-inoculation, a 62-66% *Loa loa* L3 recovery rate could be achieved in the different mouse strains. Gender of mice, type of inoculation (s.c. or i.p.) or time point of analysis (2-63 days post inoculation) did not interfere with the success of L3 recovery. In addition, administration of the immune suppressants hydrocortisone, prednisolone and cyclophosphamide did not restore *M. perstans* L3 recovery rates.

**Discussion:**

These findings show that RAG2IL-2Rg^−/−^BALB/c and C57BL/6, IL-4Rα/IL-5^−/−^ BALB/c and NSG mice were not susceptible to *M. perstans* and *O. volvulus* L3 inoculation using the applied methods, whereas *Loa loa* infection could be maintained. Further studies should investigate if humanized immunocompromised mice might be susceptible to *M. perstans*. and *O. volvulus*.

## Introduction

The human filariae *Mansonella perstans, Loa loa* and *Onchocerca volvulus* affect more than 240 million individuals. Whereas *Loa loa* is only prevalent in Western and Central Africa, *M. perstans* and *O. volvulus* are endemic throughout Sub-Saharan Africa and parts of South and Central America (1, 2). All three parasites are vector-borne diseases and transmission depend on *Culicoides* midges for *M. perstans* (3, 4), *Chrysops* flies for *Loa loa* (5) and *Simulium* black flies for *O. volvulus* (6). These vectors transmit infective stage 3 larvae (L3), which develop into adult worms that reside in body cavities (*M. perstans*) (7), subcutaneous tissue (*Loa loa*) (8) and subcutaneous nodules (*O. volvulus*) (9). Fertile female adult worms produce the microfilariae (MF) that circulate in the peripheral blood (*M. perstans* and *Loa loa*) (7, 8) or subcutaneous tissue (*O. volvulus*) (10), which can be taken up by the corresponding vectors during another blood meal. Whereas no distinct severe clinical symptoms have been associated with *M. perstans* (1), loiasis is associated with Calabar swelling, pruritis, arthralgia and eye worm (11) and onchocerciasis can cause papular dermatitis, skin hyperpigmentation or depigmentation (leopard skin) and vision loss (12, 13). Nevertheless, the majority of filarial infections remain asymptomatic due to the strong modulation of the host immunity which promotes survival of the parasites (14–16). The asymptomatic nature of filarial infections has resulted in a shortfall of knowledge, but the understanding of the biology of the nematodes and their evasion tactics within the host is important to fulfil the goals of control and elimination programmes. Thus, preclinical models are essential to study the biology of filariae and to test novel anti-filarial drugs. Although, the murine model of filariasis *Litmosoides sigmodontis* is widely used for drug screening (17), results from this model cannot be directly translated to human filarial infections. In recent years, *in vitro* culture models for *M. perstans* (18–20), *Loa loa* (21–23), and *O. volvulus* (24, 25) have been established. However, *in vitro* models do not take into account the complexity of the different niches, in which the parasite resides, and the internal environment, which is important that a drug can reach its target. Thus, *in vivo* rodent models for *Loa loa* and *O. volvulus* have been established using immunocompromised rodents (26–30), whereas no *in vivo* model for *M. perstans* has been implemented until now. Since RAG2- (27, 28, 31, 32) and IL-4/5-deficient (33–35), as well as NOD scid gamma mouse (NSG) strains (26, 36), have been proven to enhance susceptibility to filarial infections, we elucidate if *M. perstans* infections can be maintained in these mouse strains in comparison to *Loa loa* and *O. volvulus* infection.

## Methods

### Ethics

Ethical clearance was obtained from the National Institutional Review Board, Yaoundé (REF: N° 2022/12/1506/CE/CNERSH/SP) and administrative clearance from the Delegation of Public Health, South West Region (Re: R11/MINSANTE/SWR/RDPH/PS/259/382) after approval of the protocol. Special consideration was taken to minimize any health risks to the participant and involvement was strictly voluntary. The objectives of the study were explained in detail to each individual willing to participate after which they signed an informed consent form. The participant’s documents were given a code to protect the privacy of the study subject. *O. volvulus* and *M. perstans* microfilariae+ volunteers were followed up with 200mg doxycycline daily for 28 days, while albendazole 400mg daily for three weeks was administered to *L. loa* MF+ volunteers (37).

### Animals

In this study, female and male IL-4Rα/IL-5-deficient BALB/c, Rag2^tm1Fwa^II2rg^tm1Wjl^RAG2IL-2Rγ-deficient BALB/c and C57BL/6 (RAG2IL-2Rγ^−/−^), and NOD.Cg-Prkdc^scid^ Il2rg^tm1Wjl^/SzJ (NOD scid gamma, NSG) BALB/c mice were used. These mouse strains were provided by the Institute of Medical Microbiology, Immunology and Parasitology (IMMIP), University Hospital Bonn (UKB) in Germany. RAG2IL-2Rγ^−/−^ mice were purchased from Taconic Biosciences Inc (Cologne, Germany) and the other two strains were bred at the IMMIP and UKB animal facilitates, but originally, IL-4Rα/IL-5^−/−^ were a gift from Prof. Dr. Klaus Matthaei (Matthaei, Stem Cell & Gene Targeting Laboratory, ANU College of Medicine, Biology and Environment, Canberra, Australia) and NSG mice were purchased from Jackson Laboratory (Bar Harbor, Maine, USA). Mice were kept under SPF conditions following German animal protection laws and EU guidelines 2010/63/E4 and had access to food and water ad libitum. Mice were shipped in filter topped boxes to the Research Foundation for Tropical Diseases and the Environment (REFOTDE), Buea, Cameroon in agreement with the veterinary office in Bonn, Germany. Upon arrival, mice were housed in the animal facility of the Research Foundation for Tropical Diseases and the Environment, University of Buea under SPF conditions following Cameroonian animal protection laws.

### Parasite material

To infect the different mouse strains with the filarial species, infective stage 3 larvae (L3) were obtained from different batches of the collected vectors as previously described (18, 25, 33). In short, *Culicoides* midges (*M. perstans*), *Simulium damnosum* (*O. volvulus*) and *Chrysops silacea* (*Loa loa*) were collected upon a blood meal on microfilariae positive volunteers (at least 2 volunteers per filarial species). The blood-feed vectors were maintained in captivity under controlled conditions for up to 14 days to allow the development of L3. Then, L3 were isolated in RPMI-1640 medium (Sigma-Aldrich, Munich, Germany) supplemented with a 2% antibiotic cocktail (penicillin-streptomycin-neomycin; Thermo Fisher Scientific, Schwerte, Germany) by dissecting the head, the thorax and the abdomen allowing the migration of L3 into the dissecting medium. Finally, L3 were washed twice in RPMI-1640 medium to get rid of fly debris and counted using a dissecting microscope (Leica, Wetzlar, Germany). Then, the motility of the isolated L3 was assessed. Only motile L3 were considered viable and directly used for the inoculation of the different immunocompromised mouse strains.

### Experimental mouse infections

Mice were infected with the isolated alive L3 subcutaneously (s.c.) or intraperitoneally (i.p.) and the efficiency of L3 inoculation was confirmed by flushing the needle and investigating the flushed-out fluid under the microscope. Furthermore, subcutaneous implantation was performed during some inoculation experiments. In detail, mice were s.c. anaesthetized by a combination of 1μg/kg ketamine (WDT, Garbsen, Germany) and 10μg/kg medetomidine (Orion Pharma, Espoo, Finland) in combination with prophylactic antibiotic penicillin G (Sigma-Aldrich). Anaesthetised mice were arranged on sterile drapes on top of heat pads and shaved on the upper left abdomen that were swabbed with iodine (Ecolab, Monheim am Rhein, Germany). Then, a small incision was performed through the skin with sterile surgical instruments and *M. perstans* L3 worms were implanted into the subcutaneous cavity with 200μl RPMI medium (Sigma-Aldrich). Finally, mice were sutured through the skin and an α2-antagonist (atipamezole; Orion Pharma) was administered. Until recovery mice were placed on heat pads and finally placed back into IVC cages.

### Mouse dissection and worm recovery

Upon 2-63 days post-infection (p.i.) mice were euthanized by exposure to increasing concentrations of CO_2_. Cardiac blood was collected by cardiac puncture and stored in non-heparinised 1.5 ml microcentrifuge tubes to obtain sera, which was stored at -20°C. Then, heart, lung, intestine, skin and muscles were gently excised and placed in separate Petri dishes containing RPMI-1640 medium (Sigma-Aldrich) supplemented with 2% antibiotic cocktail (penicillin-streptomycin-neomycin; Thermo Fisher Scientific). Muscle tissues were teased gently to ease worm migration into the medium. Then, all tissues were incubated at 37°C for 2h to allow migration of the parasites from tissues into the medium. Finally, Petri dishes were then observed under a dissecting microscope (Leica, Wetzlar, Germany) for the presence of parasites.

### Administration of immune suppressants

The used immunocompromised mouse strains lack crucial immune cells and cell signalling mechanisms. However, to further inhibit the immune cells and responses immune suppressants were used like the corticosteroids hydrocortisone (Vinco Pharmaceutical Ltd, Lagos, Nigeria) and prednisolone (Jenapharm GmbH & Co.KG, Jena, Germany), which inhibits inflammatory transcription factors and promotes anti-inflammatory genes (37), and cyclophosphamide (Thermo Fisher Scientific), which is an antitumor agent that triggers the death of hematopoietic stem cells leading to a loss of different immune cells like neutrophils, monocytes, B and T cells (38, 39). Hydrocortisone and prednisolone (10mg/kg) were administered intramuscular (i.m.) and i.p. respectively, one day before filarial infection, whereas cyclophosphamide was administered i.p. twice, one day before infection (150mg/kg) and 4 days p.i. (100mg/kg).

## Results

### Overview of the mouse infections with different L3 species

To establish experimental filarial infections of *M. perstans, O. volvulus* and *Loa loa*, the immunocompromised mouse strains RAG2IL-2Rγ^−/−^ (lack B, T and natural killer cells), IL-4Rα/IL-5^−/−^ (impaired IL-4/5 signalling and eosinophil activation) and NSG (lack mature lymphocytes) were used. In total, 3,250 *M. perstans*, 3,337 *O. volvulus*, and 2,720 *Loa loa* L3 were isolated to infect in total of 145 immunocompromised mice. Table 1 shows a summary of mouse strains and the number of L3 that were used in this study.

### Parasite recovery rates

Since no mouse infection of *M. perstans* has been established so far, we first inoculated the different mouse strains with *M. perstans* L3 and analysed the recovery rate of the larvae 2-63 p.i. As shown in Table 2, no L3 could be recovered from any of the mouse strains independently of the type of administration (s.c. or i.p.), number of inoculated L3 (10-123/mouse), time point of analysis (2-63 days p.i.) or sex of mice. Similarly, no *O. volvulus* L3 could be recovered from the different immunocompromised mouse strains 2-7 days p.i. (Table 3).

Since no L3 could be recovered from *M. perstans* and *O. volvulus* inoculated mice, we were wondering if the isolated L3 were suitable for mouse infection experiments. However, previous studies showed that immunocompromised mice were susceptible to *Loa loa* L3 (27–29). Thus, we isolated *Loa loa* L3 from engorged Chrysops flies to inoculate the different immunocompromised mice with *Loa loa* L3. Indeed, we revealed that *Loa loa* L3 inoculation led to a recovery rate of L3 from 66% in RAG2IL-2Rγ^−/−^ C57BL/6, 65.7% RAG2IL-2Rγ^−/−^ BALB/c, and 61.8% in NSG BALB/c mice upon 2-7 days p.i., independently of sex of mice or number of inoculated L3 (Table 4). These results show that the isolated L3 were suitable to infect immunocompromised mice, but highlight that *M. perstans* and *O. volvulus* L3 did not survive in the immunocompromised mouse strains using the applied methods.

Since no L3 larvae could be recovered from *M. perstans* and *O. volvulus* L3 inoculated mice, we test if further immune suppression will increase susceptibility to *M. perstans* and *O. volvulus*. Therefore, we treated RAG2/IL-2Rγ^−/−^ mice with hydrocortisone, prednisolone and cyclophosphamide and analysed L3 recovery rate 2-7 days p.i., but again no L3 could be obtained independently of the number of inoculated larvae or sex of mice (Table 5). Since hydrocortisone, prednisolone and cyclophosphamide treatment did not improve parasite recovery in RAG2/IL-2Rγ^−/−^ mice, we consequently did not proceed with this approach with the other mouse strains to fulfil the 3R principles, especially the reduction of mouse numbers.

In conclusion, these findings suggest that RAG2IL-2Rγ^−/−^BALB/c and C57BL/6, IL-4Rα/IL-5^−/−^ BALB/c and NSG mice were not susceptible to *M. perstans* and *O. volvulus* L3 inoculation using the applied protocols and methods, whereas *Loa loa* infection can be established in these immunocompromised mouse strains.

## Discussion

Research about human filarial infections is often hindered due to the complex life cycle and difficulties in obtaining the life stages of the parasites. Thus, the mouse model of human filariasis, *Litomosoides sigmodontis*, is a suitable tool to investigate the biology of filarial infections (40, 41) and filarial-driven immune modulation of the host (42), and can be used as a preclinical model for drug testing (17). Nevertheless, findings obtained from the murine model of filariasis cannot be directly translated to the human situation.

Thus, *in vitro* models of lymphatic filariasis (43, 44), onchocerciasis (24, 25, 45), loiasis (21–23) and mansonelliosis (18–20) have been established to investigate the biology of the filarial nematodes and screen for anti-filarial drugs. Although long-term cultivation of the parasites and even the development of larvae to young adults have been achieved in some of these *in vitro* culture systems (19, 24, 25, 45), the development of L3 into fertile adult worms and consequently the production of microfilariae has not been achieved until now. However, *in vitro* filarial cultures are important for initial drug screening, but the findings need to be taken with caution due to variation in stage- and species-specific expression of filarial drug targets and possible involvement of tissue-specific responses and host immunity, which cannot be depicted *in vitro* culture systems. Thus, *in vivo* models for human filariae are needed and rodent models using immunocompromised animals have been established for *Loa loa, O. volvulus* and *Brugia* spp. (26–30, 46), but not for *Mansonella* spp.

Therefore, we aim to establish a murine model of *M. perstans* based on the knowledge of the established animal models of onchocerciasis, lymphatic filariasis and loiasis. Immunocompromised mouse strains that lack adaptive immunity pathways like RAG2^−/−^ (27, 28), IL-4/5^−/−^ (32) and NSG strains (26, 36) have been proven suitable for human filarial infections and recently, NSG mice have been also proven to be susceptible to infection with the dog heartworm *Dirofilaria immits* (47, 48). Indeed, a 62-66% L3 recovery rate could be obtained upon *Loa loa* L3 inoculation, whereas *M. perstans* and *O. volvulus* L3 were absent in all immunocompromised mouse strains. This was independent of the route of administration (i.p. or s.c.), gender of the mice, or day of analysis (day 2-63 p.i.). In addition, subcutaneous implantation of 50 *M. perstans* L3/mouse was performed in 3 NSG BALB/c mice, but also no L3 could be recovered upon 2-7 days p.i. Moreover, inhibition of immune responses by immune suppressants, which have been shown to promote *Loa loa* survival in BALB/c mice (4), did not increase susceptibility to *M. perstans* or *O. volvulus* L3. Indeed, previous studies already revealed that several immunologically intact mouse strains were not susceptible to *O. volvulus* L3 (49) and only transplantation of adult worms into SCID mice allowed worm survival for more than 20 weeks (36). It seemed that *O. volvulus* L3 can only survive in chimpanzees or mangabey monkeys (50–54), but recently it has been shown that NSG mice humanized with human immune cells allowed survival and maturation of *O. volvulus* L3 into L4 within 12 weeks of infection (26). Of note, low recovery rates (1.7-2.3%) of *O. volvulus* parasites have been also observed 4-8 weeks upon infection in non-humanized NSG mice (26), which could not be confirmed here. This might be because of the different analysis time points (2-7 days p.i. vs 4-8 weeks p.i.) and applied recovery methods, which do not include qPCR approaches and overnight incubation of muscle, skin and organs into RPMI. We suggest that increased incubation time and application of molecular approaches will increase the L3 recovery rate and detection sensitivity of the parasites, respectively and thus should be applied in future experiments. Nevertheless, parasite recovery rates increased 3-4 times when NSG mice were humanized with multiple human cell lines (26). Indeed, the addition of feeder cells that provide nutrition and support development and survival through the secretion of different factors has been proven to be important for the growth, survival and development of filariae *in vitro* (18–25, 44, 45). Humanized rodent models open up a novel perspective to establish human filarial infections not only to screen for novel drugs but also to investigate human immune cells and their immune responses towards the parasite *in vivo*, which cannot be compiled with *in situ* and *in vitro* restimulation experiments (55). Nevertheless, the question remains why *Loa loa* L3 infections can be established in the different immunocompromised mouse strains using the applied methods that were based on previously published studies (5, 23, 28, 33, 55), whereas *M. perstans* and *O. volvulus* infection were not successful. We doubt that the applied medium including antibiotics that have been used for the L3 isolation might be the cause of this phenomenon, since this medium has been also used for the successful *Loa loa* infection experiments and long-term *M. perstans* L3 *in vitro* cultures (18–20), showing that the medium does not interfere with the viability of the isolated L3. One explanation could be that *Loa loa* does not harbor *Wolbachia* endosymbionts (56, 57), which activate innate and adaptive immune responses and inflammatory pathways (58, 59) and thus can limit infection efficacy (60). Although the used immunocompromised mice lack important adaptive immune cells and signalling pathways, innate immune responses can be initiated by the *Wolbachia* endosymbionts from *M. perstans* (61) and *O. volvulus* (62) leading to the observed resistance of the mouse strains towards these filarial nematodes. In addition, filarial infections need human metabolites, cells or tissue for development and indeed, it has been shown that threonine transport from the human host is essential for *O. volvulus* but not for *Loa loa*, which can produce its own threonine (63). To overcome these obstacles humanized immunocompromised mouse model might fill the gap of the missing human factors in rodents and promising results with *O. volvulus* L3 inoculation in humanized SCID mice (26) highlight that future studies should use humanized NSG mice to establish a mouse model of *M. perstans* and *O. volvulus*. In addition, Mongolian gerbils (*Meriones unguiculatus*) are susceptible to the human filarial nematode *Brugia malayi* (64) and thus might be also considered as an alternative for future infection experiments.

## Figures and Tables

**Table 1:** Overview of number of immunocompromised mice and inoculated L3.

Filarial spp	Mouse strains	Number of infected mice	Number of inoculated L3
*M.* * * *perstans*	RAG2IL- 2Rγ^−/−^^ ^C57BL/6	18	948
	RAG2IL- 2Rγ^−/−^ BALB/c	17	1,137
	IL-4Rα/IL- 5^−/−^BALB/c	10	142
	NSG BALB/c	25	1,023
	**Total**	**70**	**3,250**
*O. * *volvulus*	RAG2IL- 2Rγ^−/−^ C57BL/6	17	1,197
	RAG2IL- 2Rγ^−/−^^ ^BALB/c	17	1,332
	NSG BALB/c	11	808
	**Total**	**45**	**3,337**
*Loa* * loa*	RAG2IL- 2Rγ^−/−^^ ^C57BL/6	10	926
	RAG2IL- 2Rγ^−/−^^ ^BALB/c	10	825
	NSG BALB/c	10	969
	**Total**	**30**	**3,250**
**Grand** **total**		**145**	**9,837**

**Table 2:** Summary of *M. perstans* L3 inoculation experiments and L3 recovery rates from the different mouse strains (F, Female; M, Male; s.c., subcutaneous; i.p., intraperitoneal).

Mouse strain	Mouse number	Sex	Method of inoculation	Days post infection	Number of inoculated L3	Number of L3 recovery	Percentage of L3 recovery
**RAG2IL** **–** **2Rγ^−^^/^^−^** **C57BL/6**	1	F	s.c.	55	55	0	0
	2	F	s.c.	48	48	0	0
	3	F	s.c.	48	48	0	0
	4	F	s.c.	45	45	0	0
	5	F	s.c.	53	53	0	0
	6	F	s.c.	35	35	0	0
	7	F	s.c.	35	35	0	0
	8	F	s.c.	6	6	0	0
	9	F	s.c.	2	2	0	0
	10	F	s.c.	2	2	0	0
	11	M	i.p.	2	2	0	0
	12	M	i.p.	5	5	0	0
	13	M	i.p.	5	5	0	0
	14	M	i.p.	7	7	0	0
	15	M	i.p.	7	7	0	0
	**Total**				**212**	**0**	**0**
**RAG2IL – 2Rγ^−/−^ BALB/c**	1	M	s.c.	7	102	0	0
	2	M	s.c.	7	100	0	0
	3	M	s.c.	7	81	0	0
	4	M	s.c.	5	86	0	0
	5	F	s.c.	5	75	0	0
	6	F	s.c.	2	66	0	0
	7	F	s.c.	2	105	0	0
	**Total**				**615**	**0**	**0**
**IL - 4Rα/IL - 5^−/−^ BALB/c**	1	M	s.c.	63	13	0	0
	2	M	s.c.	51	17	0	0
	3	M	s.c.	52	14	0	0
	4	M	s.c.	40	10	0	0
	5	M	s.c.	35	14	0	0
	6	M	i.p.	33	11	0	0
	7	M	i.p.	27	13	0	0
	8	M	i.p.	27	13	0	0
	9	M	i.p.	2	20	0	0
	10	M	i.p.	2	17	0	0
	**Total**				**142**	**0**	**0**
**NSG BALB/c**	1	M	s.c.	2	50	0	0
	2	M	s.c.	2	42	0	0
	3	F	s.c.	2	59	0	0
	4	F	s.c.	2	41	0	0
	5	M	s.c.	5	51	0	0
	6	M	s.c.	5	50	0	0
	7	M	s.c.	5	55	0	0
	8	F	s.c.	5	70	0	0
	9	F	s.c.	7	73	0	0
	10	F	s.c.	7	50	0	0
	11	M	s.c.	7	66	0	0
	12	M	s.c.	7	75	0	0
	**Total**				**682**	**0**	**0**

**TABLE 3:** Summary of *O. volvulus* L3 inoculation experiments and L3 recovery rates from the different mouse strains (F, Female; M, Male; s.c., subcutaneous).

Mouse strain	Mouse number	Sex	Method of inoculation	Days post infection	Number of inoculated L3	Number of L3 recovery	Percentage of L3 recovery
**RAG2IL – 2Rγ^−/−^ C57BL/6**	1	F	s.c.	2	50	0	0
	2	F	s.c.	2	50	0	0
	3	F	s.c.	5	50	0	0
	4	F	s.c.	5	50	0	0
	5	M	s.c.	5	50	0	0
	6	M	s.c.	7	50	0	0
	7	M	s.c.	7	58	0	0
	8	M	s.c.	7	63	0	0
	**Total**				**421**	**0**	**0**
**RAG2IL – 2Rγ^−/−^ BALB/c**	1	M	s.c.	2	50	0	0
	2	M	s.c.	2	50	0	0
	3	M	s.c.	2	50	0	0
	4	M	s.c.	5	50	0	0
	5	F	s.c.	5	50	0	0
	6	F	s.c.	5	55	0	0
	7	F	s.c.	7	58	0	0
	8	F	s.c.	7	54	0	0
	**Total**				**417**	**0**	**0**
**NSG BALB/c**	1	F	s.c.	2	50	0	0
	2	F	s.c.	2	50	0	0
	3	F	s.c.	2	50	0	0
	4	F	s.c.	2	62	0	0
	5	F	s.c.	5	60	0	0
	6	M	s.c.	5	43	0	0
	7	M	s.c.	5	93	0	0
	8	M	s.c.	5	100	0	0
	9	M	s.c.	7	100	0	0
	10	M	s.c.	7	100	0	0
	11	F	s.c.	7	100	0	0
	**Total**				**808**	**0**	**0**

**TABLE 4:** Summary of *Loa loa* L3 inoculation experiments and L3 recovery rates from the different mouse strains (F, Female; M, Male; s.c., subcutaneous).

Mouse strain	Mouse number	Sex	Method of inoculation	Days post infection	Number of inoculated L3	Number of L3 recovery	Percentage of L3 recovery
**RAG2IL – 2Rγ – / – C57BL/6**	1	M	s.c.	2	100	70	70.0
	2	M	s.c.	2	100	65	65.0
	3	M	s.c.	2	100	72	72.0
	4	F	s.c.	2	100	66	66.0
	5	F	s.c.	5	100	77	77.0
	6	F	s.c.	5	100	65	65.0
	7	F	s.c.	5	93	40	43.0
	8	F	s.c.	7	89	55	61.8
	9	F	s.c.	7	75	63	84.0
	10	F	s.c.	7	69	38	55.1
	**Total**				**926**	**611**	**66.0**
**RAG2IL – 2Rγ – / – BALB/c**	1	F	s.c.	2	58	40	69.0
	2	F	s.c.	2	62	45	72.6
	3	F	s.c.	2	55	32	58.2
	4	M	s.c.	5	50	36	72.0
	5	M	s.c.	5	100	45	45.0
	6	M	s.c.	5	100	60	60.0
	7	M	s.c.	5	100	66	66.0
	8	M	s.c.	7	100	78	78.0
	9	M	s.c.	7	100	75	75.0
	10	M	s.c.	7	100	65	65.0
	**Total**				**825**	**542**	**65.7**
**NSG BALB/c**	1	M	s.c.	2	100	56	56.0
	2	M	s.c.	2	63	44	69.8
	3	M	s.c.	2	55	18	32.7
	4	F	s.c.	5	100	85	85.0
	5	F	s.c.	5	110	68	61.8
	6	F	s.c.	5	120	73	60.8
	7	F	s.c.	7	121	66	54.5
	8	F	s.c.	7	100	74	74.0
	9	F	s.c.	7	100	52	52.0
	10	F	s.c.	7	100	63	63.0
	**Total**				**969**	**599**	**61.8**

**TABLE 5:** Summary of *M. perstans* and *O. volvulus* L3 recovery rates from the RAG2/IL-2Rγ^−/−^ C57BL/6 and BALB/c mice (F, Female; M, Male; L3 larvae were inoculated subcutaneously).

Mouse strain	Mouse number	Sex	Parasite	Days postinfection		Number of inoculated L3	Number of L3 recovery	Percentage of L3 recovery
**RAG2IL – 2Rγ – / – C57BL/6**	**Hydrocortisone**							
	1	F	*M.* * * *perstans*	2		50	0	0
	2	F		5		46	0	0
	3	F		5		89	0	0
	**Total**					**185**	**0**	**0**
	1	M	*O. * *volvulus*	5		100	0	0
	2	M		5		100	0	0
	3	M		2		58	0	0
	**Total**					**258**	**0**	**0**
	**Prednisolone**							
	1	M	*M.* * * *perstans*	2		100	0	0
	2	M		2		90	0	0
	3	F		5		50	0	0
	4	F		7		55	0	0
	**Total**					**295**	**0**	**0**
	1	F	*O. * *volvulus*	5		100	0	0
	2	F		5		100	0	0
	3	F		2		65	0	0
	**Total**					**265**	**0**	**0**
	**Cyclophosphamide**							
	1	M	*M.* * perstans*		5	40	0	0
	2	M			5	40	0	0
	3	M			5	33	0	0
	**Total**					**113**	**0**	**0**
	1	F	*O. * *volvulus*		5	100	0	0
	2	F			5	78	0	0
	3	F			7	75	0	0
	**Total**					**253**	**0**	**0**
**RAG2IL – 2Rγ –/ – BALB/c**	**Hydrocortisone**							
	1	M	*M.* * perstans*		2	56	0	0
	2	M			2	61	0	0
	3	F			5	74	0	0
	4	F			5	33	0	0
	**Total**					**224**	**0**	**0**
	1	M	*O. * *volvulus*		2	100	0	0
	2	M			5	100	0	0
	3	M			5	75	0	0
	**Total**					**275**	**0**	**0**
	**Prednisolone**							
	1	F	*M.* * perstans*		2	48	0	0
	2	F			2	20	0	0
	3	F			5	67	0	0
	4	M			5	50	0	0
	**Total**					**185**	**0**	**0**
	1	M	*O. * *volvulus*		2	100	0	0
	2	M			2	100	0	0
	3	F			5	120	0	0
	**Total**					**320**	**0**	**0**
	**Cyclophosphamide**							
	1	M	*M.* * perstans*		5	40	0	0
	2	M			5	40	0	0
	3	M			5	33	0	0
	**Total**					**113**	**0**	**0**
	1	F	*O. * *volvulus*		5	100	0	0
	2	F			5	108	0	0
	3	F			7	112	0	0
	**Total**					**320**	**0**	**0**

## Data Availability

The original contributions presented in the study are included in the article/supplementary material. Further inquiries can be directed to the corresponding author.
